# Discordant ^18^F-FES PET and ER immunohistochemistry in ER-positive bone metastasis: a potential imaging pitfall

**DOI:** 10.1007/s00259-026-08019-6

**Published:** 2026-06-27

**Authors:** Anne-Claire Dupont, Lucas Gaouaou, Anne-Marie Bergemer-Fouquet, Marie-Agnès By, Maria-Joao Santiago-Ribeiro

**Affiliations:** 1 Unité de Radiopharmacie, CHRU de Tours, Tours, France; 2 UMR 1253, iBrain, Université de Tours, Inserm, Tours, France; 3 Service de Médecine Nucléaire, CHRU de Tours, Tours, France; 4 Service d’Anatomie et Cytologie pathologiques, CHRU de Tours, Tours, France; 5 Service d’Oncology, CHRU de Tours, Tours, France

A 48-year-old woman with a history of estrogen receptor-positive (ER+), HER2-negative breast cancer treated with surgery, chemotherapy, radiotherapy, and endocrine therapy (Tamoxifen) presented with sternal pain four years after initial diagnosis. ^18^F-FDG PET/CT (A) demonstrated a hypermetabolic sternal lesion suspicious for metastatic disease. A corresponding osteolytic lesion of the sternum was identified on CT imaging. In contrast, ^18^F-FES PET/CT (B) was performed after a 6-week discontinuation of tamoxifen, with no other endocrine therapy administered during the washout period, and showed no tracer uptake in the same lesion. Subsequent biopsy confirmed metastatic breast carcinoma with preserved ER expression (C). Histopathology revealed tumor infiltration on hematoxylin–eosin staining and strong ER positivity on immunohistochemistry. This discordance illustrates a key limitation of ^18^F-FES imaging: tracer uptake reflects functional receptor availability rather than receptor expression [1, 2]. Potential explanations for the absence of FES uptake despite preserved ER expression include persistent receptor occupancy, altered receptor functionality, tumor heterogeneity, impaired ligand-binding capacity, or other biological factors affecting tracer binding. However, the precise mechanism underlying this discordance cannot be determined from this single case [3].

The case highlights a potential pitfall in interpreting FES PET, particularly in previously treated patients. A negative FES scan should not be interpreted as absence of ER expression, especially when clinical or metabolic findings are suspicious. Recognition of this limitation is essential to avoid misclassification of ER status and inappropriate therapeutic decisions. Multimodal correlation, including FDG PET and histopathology, remains critical in such discordant cases (Fig. 1).

**Fig. 1:**
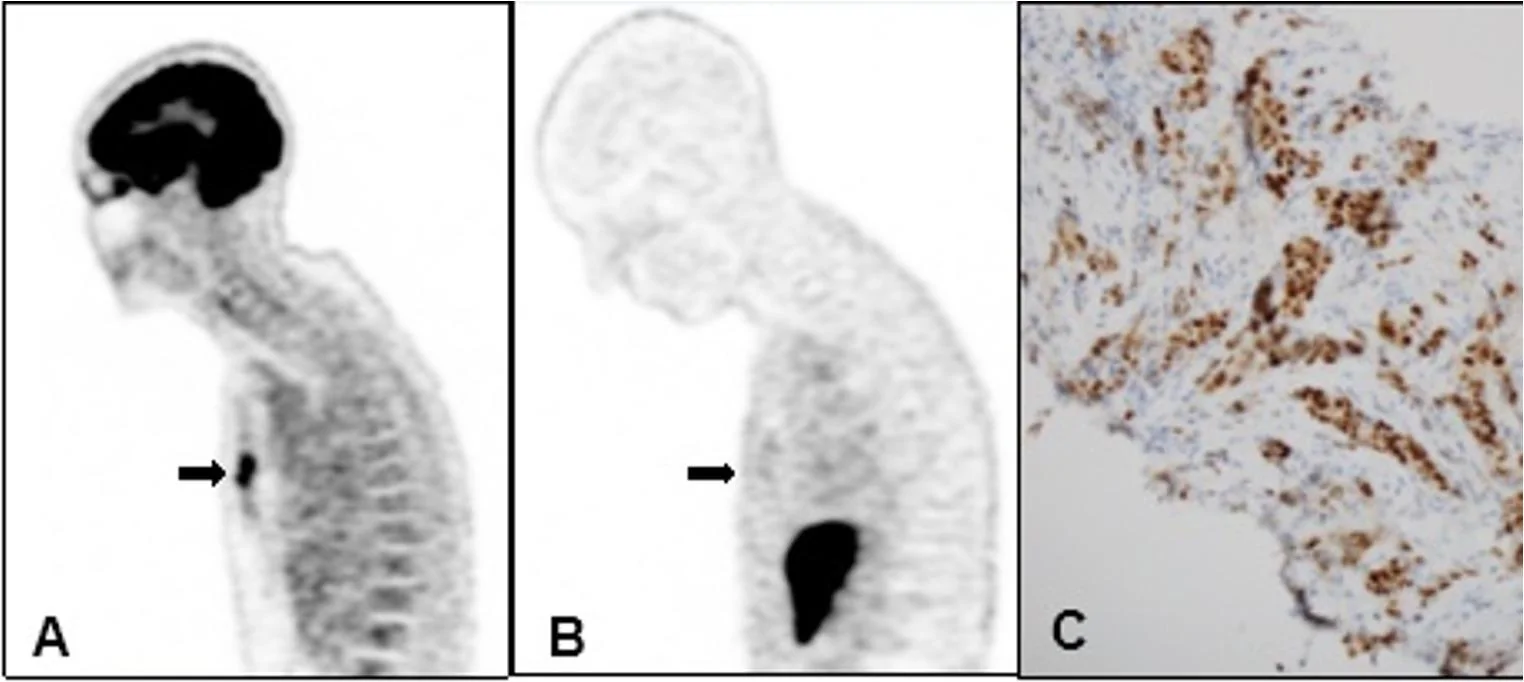
Multimodal imaging and histopathological correlation of sternal metastasis. (**A**) 18F-FDG PET/CT demonstrating intense hypermetabolic activity in the sternal lesion, consistent with metabolically active metastasis. (**B**) 18F-FES PET/CT performed approximatively 2 months later, following a 6-week discontinuation of endocrine therapy, showing no significant tracer uptake in the sternal lesion. (**C**) Immunohistochemistry of the sternal biopsy demonstrating positive estrogen receptor (ER) expression in tumor cells

## References

[1] Kurland BF, Wiggins JR, Coche A, Fontan C, Bouvet Y, Webner P, et al. Whole-body characterization of estrogen receptor status in metastatic breast cancer with 16α-18F-Fluoro-17β-estradiol positron emission tomography: meta-analysis and recommendations for integration into clinical applications. Oncologist 2020;25(10):835–44. 10.1634/theoncologist.2019-0967 PMC7543360 32374053

[2] Peterson LM, Kurland BF, Link JM, Schubert EK, Stekhova S, Linden HM, et al. Factors influencing the uptake of 18F-fluoroestradiol in patients with estrogen receptor positive breast cancer. Nucl Med Biol 2011;38(7):969–78. 10.1016/j.nucmedbio.2011.03.002 PMC4108284 21982568

[3] van Kruchten M, de Vries EGE, Brown M, de Vries EFJ, Glaudemans AWJM, Dierckx Rajo, et al. PET imaging of oestrogen receptors in patients with breast cancer. Lancet Oncol 2013;14(11):e465–75. 10.1016/S1470-2045(13)70292-4 24079874

